# Outcomes of thoracic aortic interventions in Marfan syndrome in the state of Texas over 11 years

**DOI:** 10.1093/icvts/ivad128

**Published:** 2023-08-16

**Authors:** Matthew F Mikulski, Andrew Well, Carlos M Mery, Gregory Johnson, Erin A Gottlieb, Charles D Fraser, Ziv Beckerman

**Affiliations:** Department of Surgery and Perioperative Care, Dell Medical School, The University of Texas at Austin, Austin, TX, USA; Texas Center for Pediatric and Congenital Heart Disease, UT Health Austin and Dell Children’s Medical Center, Austin, TX, USA; Department of Surgery and Perioperative Care, Dell Medical School, The University of Texas at Austin, Austin, TX, USA; Texas Center for Pediatric and Congenital Heart Disease, UT Health Austin and Dell Children’s Medical Center, Austin, TX, USA; Department of Surgery and Perioperative Care, Dell Medical School, The University of Texas at Austin, Austin, TX, USA; Texas Center for Pediatric and Congenital Heart Disease, UT Health Austin and Dell Children’s Medical Center, Austin, TX, USA; Texas Center for Pediatric and Congenital Heart Disease, UT Health Austin and Dell Children’s Medical Center, Austin, TX, USA; Department of Pediatrics, Dell Medical School, The University of Texas at Austin, Austin, TX, USA; Department of Surgery and Perioperative Care, Dell Medical School, The University of Texas at Austin, Austin, TX, USA; Texas Center for Pediatric and Congenital Heart Disease, UT Health Austin and Dell Children’s Medical Center, Austin, TX, USA; Department of Surgery and Perioperative Care, Dell Medical School, The University of Texas at Austin, Austin, TX, USA; Texas Center for Pediatric and Congenital Heart Disease, UT Health Austin and Dell Children’s Medical Center, Austin, TX, USA; Department of Surgery and Perioperative Care, Dell Medical School, The University of Texas at Austin, Austin, TX, USA; Texas Center for Pediatric and Congenital Heart Disease, UT Health Austin and Dell Children’s Medical Center, Austin, TX, USA

**Keywords:** Marfan syndrome, Outcomes, Thoracic aortic aneurysms, Aortic dissections

## Abstract

**OBJECTIVES:**

Marfan syndrome is a heritable connective tissue disorder with significant aortopathy and conveys substantial cardiovascular morbidity. This study characterizes the mortality and morbidities of thoracic aortic interventions (TAI) in the Marfan syndrome population in the state of Texas from 2009 to 2019.

**METHODS:**

A retrospective review of the Texas Inpatient Discharge Dataset from 1 January 2009 to 31 December 2019. Discharges from acute care hospitals with a Marfan syndrome diagnosis by the International Classification of Diseases 9/10 codes and a procedure code for TAI were analysed utilizing descriptive, univariate and multivariable regression statistics.

**RESULTS:**

There were 4641 Marfan syndrome discharges identified, of whom 644 (13.9%) underwent TAI. Thoracic or thoraco-abdominal aortic dissection or rupture was noted in 223 (34.6%). Thirty-three (5.1%) had a concomitant coronary artery intervention. There were 30 (4.7%) in-hospital mortalities, 126 (19.6%) diagnoses of acute renal failure (ARF), 52 (8.1%) had mechanical ventilation >96 h and the median length of stay was 10 [interquartile range (IQR) 7–16] days. After adjustment, concomitant coronary artery intervention was associated with in-hospital mortality [odds ratio (OR) 3.69 [IQR 1.15–11.90], *P* = 0.029] and ARF (OR 2.66 [IQR 1.19–5.94], *P* = 0.017). Aortic dissections/ruptures were associated with ARF (OR 1.73 [IQR 1.14–2.63], *P* = 0.010), ventilation >96 h (OR 2.19 [IQR 1.21–3.97], *P* = 0.010), and 15% longer length of stay (95% confidence interval 2.4–29.1%, *P* = 0.038).

**CONCLUSIONS:**

TAI are frequent among the hospitalized Marfan Syndrome population. Concomitant coronary intervention is associated with increased risk of death and aortic dissections/ruptures are associated with increased morbidity. The high prevalence of aortic dissections/ruptures points to a potential target for improving imaging surveillance, adherence to treatment guidelines and preventative management of Marfan syndrome aortopathy.

**Figure:**
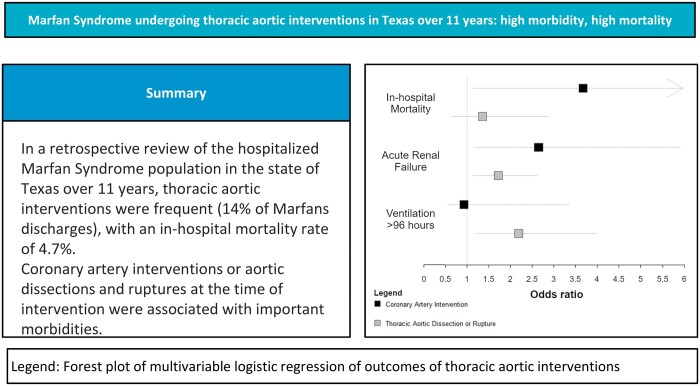

## INTRODUCTION

Marfan syndrome (MFS) is a connective tissue disorder associated with a defect in the fibrillin 1 protein [1]. It is most commonly inherited in an autosomal dominant fashion, but can be sporadic [1]. While affecting many different organ systems, it is associated with significant cardiovascular morbidity [2]. Dilation of the aortic root progressing to thoracic or thoraco-abdominal aortic dissection or rupture (TADR) was historically the leading cause of death in MFS [3]. Management of the thoracic aorta, therefore, has been researched extensively including: medical management [4–6], the Bentall procedure [7] versus aortic valve-sparing procedures [8–10], timing of aortic root surgery based on dilation and/or increases in size [11, 12], and open versus endovascular approaches [13–15]. To better track TADR outcomes, 2 registries were created: the International Registry of Acute Aortic Dissection [16] and the National Registry of Genetically Triggered Thoracic Aortic Aneurysms [17]. Both have yielded valuable insight into TADR, but they include other aortopathies, do not provide epidemiologic context such as MFS without TADR and do not capture settings in which prophylactic thoracic aortic interventions (TAI) may occur without TADR [8–12].

Despite increased awareness and research, in-hospital outcomes of TAI in the MFS population are limited. The goal of this study is to analyse contemporary outcomes of TAI in the MFS population in a large, statewide database over 11 years. The primary objective was to describe the rates of in-hospital mortality and complications such as acute renal failure (ARF), prolonged ventilation, temporary mechanical circulatory support (TMCS), including extracorporeal membranous oxygenation and longer length of stay (LOS), and evaluate factors associated with those complications.

## PATIENTS AND METHODS

### Ethical statement

Institutional Review Board oversight was waived for this study as it consisted of publicly-curated, deidentified data.

### Data source

Data originated from the Texas Inpatient Discharge Public Use File (TIDD) from 1 January 2009 to 31 December 2019. The TIDD is a deidentified, administrative database whose full details have been previously described [18, 19]. It includes discharges from almost all hospitals in the state of Texas. Exemptions include those from hospitals in a county with a population less than 35,000, or those located in a county with a population more than 35,000 and with fewer than 100 licensed hospital beds and not located in an area that is delineated as an urbanized area by the United States Bureau of the Census. The TIDD provides an admitting, principal and up to 24 other diagnoses, and a principal procedure and up to 24 other procedures for each discharge. From 2009 to 2015q3, diagnoses and procedures were coded using the International Classification of Diseases (ICD)-9. Records from 2015q4 to 2019 were coded using the 10th Revision (ICD-10).

### Study population

Discharges from acute care hospitalizations with MFS diagnoses (ICD codes 759.82, Q87.40–Q87.43) were identified. Demographics included sex, race, ethnicity, ageand insurance status. In the TIDD, patient age is categorized into 16 groups, which were collated into 0–9, 10–19, 20–34, 35–49, 50–64 and 65+years. Insurance status was grouped into Private, Uninsured, Medicare, Medicaid and Other. Clinical outcomes provided by the TIDD included in-hospital mortality and LOS. Discharges with incomplete demographic information were excluded from analysis.

TAI were identified by procedure codes indicating open surgical and endovascular interventions involving the thoracic aorta. Further diagnoses, procedures and outcomes identified using ICD-9/10 codes included: open versus endovascular approach, mitral valve interventions, aortic valve interventions and coronary artery interventions, hypertension, atherosclerosis, dyslipidaemia, diabetes mellitus, tobacco use, bicuspid aortic valves, ARF, TMCS and mechanical ventilation >96 h (MV96; Supplementary Material, Table S1). Additionally, TADR diagnoses grouped thoracic and thoraco-abdominal locations due to a lack of granularity afforded by ICD-9 codes. Hospitals were stratified into tertiles based on TAI volume.

### Statistical analysis

Descriptive statistics were used for demographics, clinical characteristics and outcomes. Categorical variables are reported as *n*(%). LOS is reported in median [interquartile range (IQR)] days. Chi-squared and Fisher’s exact test were utilized to analyse non-continuous variables. Wilcoxon signed-rank test and Kruskal–Wallis test were used for non-normally distributed comparisons. Multivariable logistic and linear regressions were utilized for categorical and continuous variables, respectively. The models included clinically-relevant variables. There were at least 10 outcomes for each variable to reduce the risk of overfitting the models. All variables included were assessed for collinearity. All statistical tests were 2-tailed and a *P*-value < 0.05 was considered significant. All statistical analyses adhered to the Journal’s statistical guidelines and were performed using R(20) and RStudio (RStudio, Boston, MA, USA) [20, 21].

## RESULTS

### Population and characteristics

A total of 4641 MFS discharges were identified, out of which there were 644 (13.9%) discharges that underwent TAI, which comprised the study group (Table 1). Of the study group, a principal diagnosis of TADR was identified in 223 (34.6%) discharges. Other principle diagnoses, stratified by age group, are shown in Table 2. TADR or thoracic aortic aneurysms were the most common cardiovascular principal diagnosis in every age group except 0–9 years.

**Table 1:** Baseline characteristics: Thoracic Aortic Intervention Cohort

Factor	TAI (*n* = 644), *n* (%)
Epidemiologic factors	
Female	248 (38.5%)
White	421 (65.4%)
Hispanic	141 (21.9%)
Paediatric (<18 years)	35 (5.4%)
Age (years)	
0–9	5 (0.8%)
10–19	48 (7.5%)
20–34	188 (29.2%)
35–49	209 (32.5%)
50–64	160 (24.8%)
65+	34 (5.3%)
Type of insurance	
Private	437 (67.9%)
Uninsured	53 (8.2%)
Medicare	51 (7.9%)
Medicaid	77 (12.0%)
Other	26 (4.0%)
Risk factors	
Hypertension	416 (64.6%)
Atherosclerosis	11 (1.7%)
Lipid disorders	78 (12.1%)
Diabetes mellitus	30 (4.7%)
Tobacco use	100 (15.5%)
Bicuspid aortic valve	16 (2.5%)
Concomitant interventions	
Mitral valve intervention	88 (13.7%)
Aortic valve intervention	119 (18.5%)
Coronary artery intervention	33 (5.1%)
TAI approach	
Endovascular	42 (6.5%)
Principal diagnosis	
TADR	223 (34.6%)
Tertiles by hospital volume	
Low volume	68 (10.6%)
Mid volume	78 (12.1%)
High volume	498 (77.3%)

TADR: thoracic and thoraco-abdominal dissections and ruptures; TAI: thoracic aortic intervention.

**Table 2:** Cardiovascular principal diagnoses with stratification and heat mapping by age groups

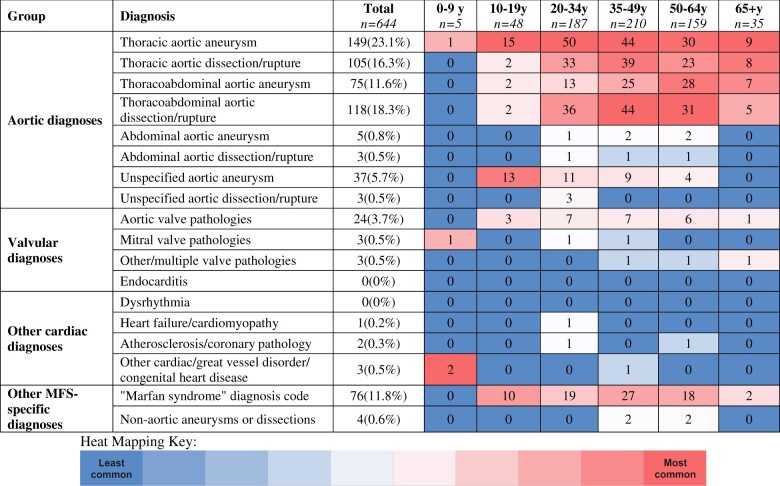

In addition to TAI, concomitant cardiac procedures included interventions on the: mitral valve (*n* = 88, 13.7%), aortic valve (*n* = 119, 18.5%) and coronary arteries (*n* = 33, 5.1%). Subgroup analysis showed 19 (21.6%) of mitral valve interventions, 32 (26.9%) of aortic valve interventions and 14 (42.4%) of coronary artery interventions took place in the setting of TADR. TAI approach was endovascular in 42 (6.5%) cases, none of which occurred in the setting of TADR**.**

### Hospitals

**Figure 1:**
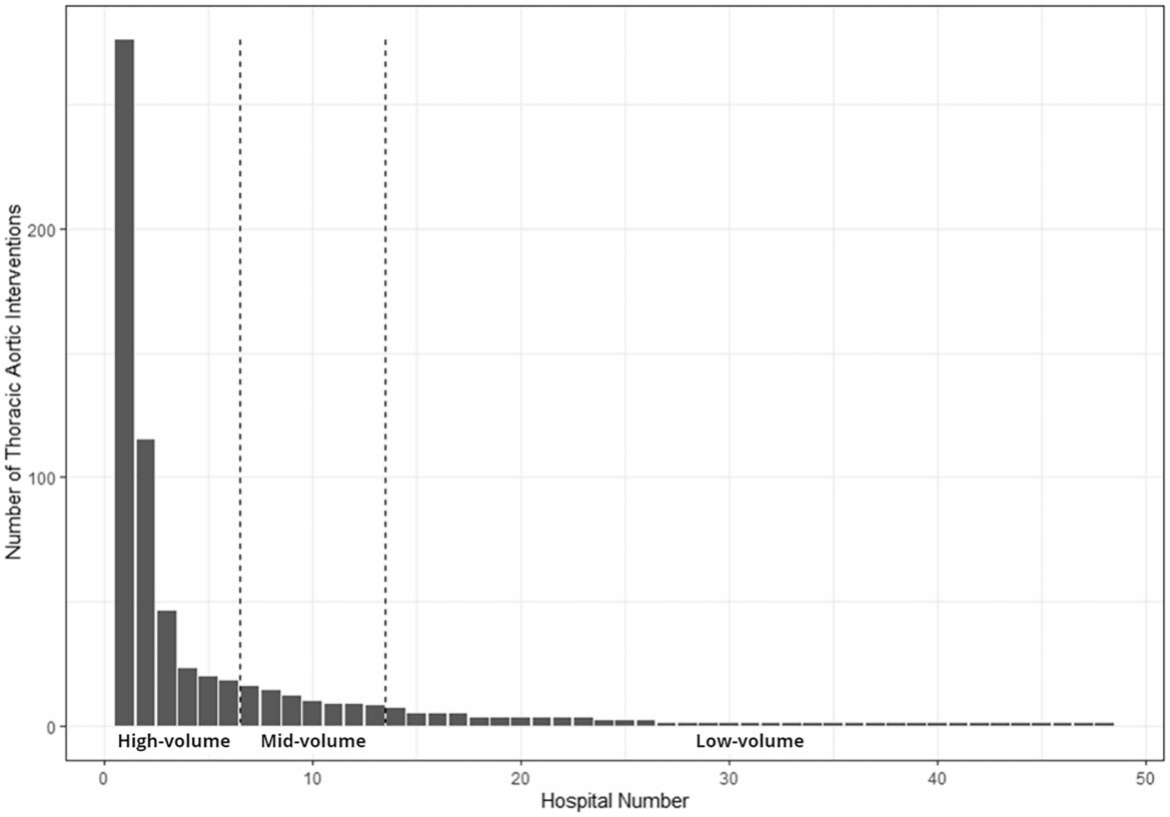
Distribution of thoracic aortic interventions by hospital. Hospitals were grouped by volume into tertiles by volume, encompassing high-volume, mid-volume and low-volume hospitals, with most procedures occurring in the high-volume tertile.

Forty-eight hospitals performed at least 1 TAI (Fig. 1). The Low-volume tertile encompassed 35 hospitals and accounted for 68 (10.6%) TAI, the mid-volume tertile included 7 hospitals accounting for 78 (12.1%) TAI, and the high-volume tertile included 6 hospitals encompassing 498 (77.3%) of TAI.

### In-hospital mortality

**Figure 2:**
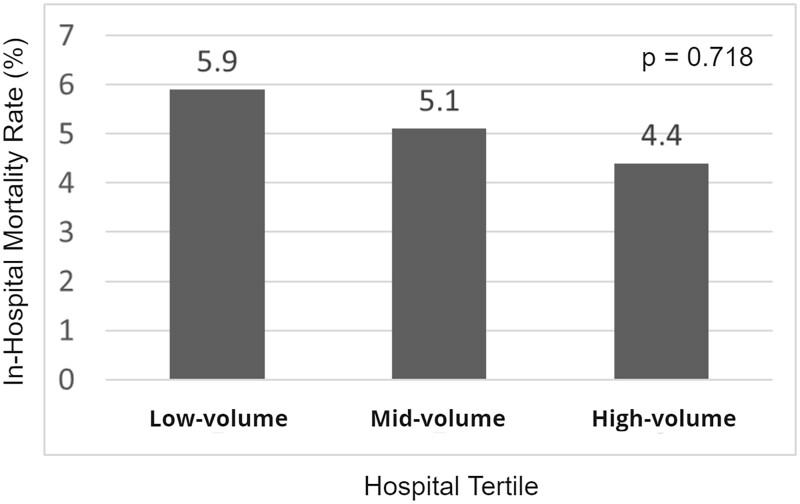
In-hospital mortality rate by high-volume, mid-volume, and low-volume hospital tertile. There was no difference in the in-hospital mortality rate between high-, medium- or low-volume hospital tertiles.

In-hospital mortality occurred in 30 (4.7%) TAI discharges (Table 3). The ages with the highest number of TAI in-hospital mortalities were 20–34 and 50–64 years with 9 (30%) each (Supplementary Material, Table S2). There was a decrease in in-hospital mortality rate as procedure volume increased by hospital tertile, but it did not amount to statistical significance (*P* = 0.718), nor did an individual assessment of the low-volume tertile compared to the high-volume tertile (*P* = 0.588; Fig. 2). Concomitant coronary artery interventions were associated with increased in-hospital mortality (*n* = 4, 13.3% vs *n* = 29, 4.7%, *P* = 0.037). After adjustment (Table 4), coronary artery interventions remained associated with in-hospital mortality {odds ratio 3.69 [95% confidence interval (CI) 1.15–11.90], *P* = 0.029}. There were no other associations identified.

**Table 3:** Univariate analysis of outcomes

	Total	In-hospital mortality (*n* = 30), *n* (%)	Alive (*n* = 614), *n* (%)	*P*-value	ARF (*n* = 126), *n* (%)	No ARF (*n* = 518), *n* (%)	*P*-value	MV96 (*n* = 52), *n* (%)	No MV96 (*n* = 592), *n* (%)	*P*-value
Hospital tertile										
Low volume	68	4 (13.3%)	64 (10.4%)	0.722	12 (9.5%)	56 (10.8%)	0.949	6 (11.5%)	62 (10.5%)	0.969
Mid volume	78	4 (13.3%)	74 (12.1%)		15 (11.9%)	63 (12.2%)		6 (11.5%)	72 (12.2%)	
High volume	498	22 (73.3%)	476 (77.5%)		99 (78.6%)	399 (77.0%)		40 (76.9%)	458 (77.4%)	
Co-morbidities										
Hypertension	416	18 (60.0%)	398 (64.8%)	0.59	86 (68.3%)	330 (63.7%)	0.338	32 (61.5%)	384 (64.9%)	0.631
Atherosclerosis	11	0 (0%)	11 (1.8%)	0.46	4 (3.2%)	7 (1.4%)	0.157	1 (1.9%)	10 (1.7%)	0.901
Lipid disorders	78	1 (3.3%)	77 (12.5%)	0.131	21 (16.7%)	57 (11.0%)	0.081	6 (11.5%)	72 (12.2%)	0.895
Diabetes mellitus	30	0 (0%)	30 (4.9%)	0.215	6 (4.8%)	24 (4.6%)	0.951	1 (1.9%)	29 (4.9%)	0.329
Tobacco use	100	5 (16.7%)	95 (15.5%)	0.86	25 (19.8%)	75 (14.5%)	0.136	10 (19.2%)	90 (15.2%)	0.442
Bicuspid aortic valve	16	0 (0%)	16 (2.6%)	0.371	1 (0.8%)	15 (2.9%)	0.174	0 (0%)	16 (2.7%)	0.23
Procedures										
Mitral valve	88	4 (13.3%)	84 (13.7%)	0.957	13 (10.3%)	75 (14.5%)	0.223	8 (15.4%)	80 (13.5%)	0.706
Aortic valve	119	3 (10.0%)	116 (18.9%)	0.22	22 (17.5%)	97 (18.7%)	0.743	9 (17.3%)	110 (18.6%)	0.821
Coronary artery	33	4 (13.3%)	29 (4.7%)	**0.037**	12 (9.5%)	21 (4.1%)	**0.013**	3 (5.8%)	30 (5.1%)	0.826
Endovascular	42	0 (0%)	42 (6.8%)	0.138	4 (3.2%)	38 (7.3%)	0.09	1 (1.9%)	41 (6.9%)	0.161
Diagnosis										
TADR	223	13 (43.3%)	210 (34.2%)	0.305	59 (46.8%)	164 (31.7%)	**0.001**	27 (51.9%)	196 (33.1%)	**0.006**

ARF: acute renal failure; MV96: mechanical ventilation >96 h; TADR: thoracic and thoraco-abdominal dissections and ruptures.

Bold values denote statistical significance.

**Table 4:** Multivariable logistic regression for in-hospital mortality

Factor	OR in-hospital mortality (95% CI)	*P*-value
Procedures		
Mitral or aortic valve intervention	0.59 (0.24–1.47)	0.256
Coronary artery intervention	3.69 (1.15–11.90)	**0.029**
Diagnosis		
TADR	1.36 (0.65–2.88)	0.417

CI: confidence interval; OR: odds ratio; TADR: thoracic and thoraco-abdominal dissections and ruptures.

Bold values denote statistical significance.

### Acute renal failure

ARF was documented in 126 (19.6%) discharges (Table 3). It most frequently occurred in the 50–64 year group (*n* = 40, 25%, *P* = 0.037) and there was a lower prevalence in females [*n* = 38 (30.2%) vs *n* = 210 (40.5%), *P* = 0.032] (Supplementary Material, Table S2). ARF was increased with coronary artery interventions [*n* = 12 (9.5%) vs *n* = 21 (4.1%) *P* = 0.013] and with a TADR diagnosis [*n* = 59 (46.8%) vs *n* = 164 (31.7%), *P* = 0.001]. After adjustment (Table 5), the 50–64 year age group had 1.99 (95% CI 1.09–3.62, *P* = 0.025) odds of ARF compared to the 20–34 year age group, females had 0.57 (95% CI 0.36–0.88, *P* = 0.011) odds, coronary artery interventions conferred 2.66 (95% CI 1.19–5.94, *P* = 0.017) odds, and TADR had 1.73 (95% CI 1.14–2.63, *P* = 0.010) odds of ARF.

**Table 5:** Multivariable logistic regression for acute renal failure

Factor	OR ARF (95% CI)	*P-*value
Demographics		
Female	0.57 (0.36–0.88)	**0.011**
White	0.79 (0.51–1.24)	0.306
Age (years)		
0–9	0.00 (0–inf)	0.983
10–19	0.56 (0.18–1.78)	0.328
20–34	Ref	–
35–49	1.78 (1.03–3.07)	**0.038**
50–64	1.99 (1.09–3.62)	**0.025**
65+	1.76 (0.67–4.64)	0.250
Insurance		
Private	Ref	–
Uninsured	1.19 (0.57–2.48)	0.639
Medicare	1.53 (0.65–3.60)	0.331
Medicaid	1.10 (0.60–2.03)	0.752
Other	1.03 (0.36–2.97)	0.950
Hypertension	1.04 (0.67–1.62)	0.861
Lipid disorders	1.21 (0.67–2.18)	0.536
Diabetes mellitus	0.82 (0.31–2.16)	0.691
Tobacco	1.26 (0.75–2.14)	0.380
Procedure		
Mitral valve	0.80 (0.41–1.55)	0.510
Aortic valve	0.91 (0.52–1.59)	0.741
Coronary artery	2.66 (1.19–5.94)	**0.017**
Diagnosis		
TADR	1.73 (1.14–2.63)	**0.010**

ARF: acute renal failure; CI: confidence interval; OR: odds ratio; TADR: thoracic and thoraco-abdominal dissections and rupture.

Bold values denote statistical significance.

### Temporary mechanical circulatory support

There were 17 (2.6%) discharges with TMCS (Supplementary Material, Table S3). Tobacco use [*n* = 6 (35.3%) vs *n* = 94 (15.0%), *P* = 0.023], coronary artery interventions [*n* = 3 (17.6%) vs *n* = 30 (4.8%), *P* = 0.018] and TADR [*n* = 10 (58.8%) vs *n* = 213 (34.0%), *P* = 0.034] were associated with increased prevalence of TMCS.

### Mechanical ventilation >96 h

There were 52 (8.1%) discharges having MV96 (Table 3). There was variation in age (*P* = 0.026) with the highest prevalence occurring in the 50- to 64-year age group (*n* = 19, 11.9%). TADR had higher prevalence of MV96 than those without [*n* = 27 (51.9%) vs *n* = 196 (33.1%), *P* = 0.006]. These associations persisted after adjustment with the 50- to 64-year age group having 2.63 (95% CI 1.17–5.94, *P* = 0.020) odds relative to the 20- to 34-year age group and TADR having 2.19 (95% CI 1.21–3.97, *P* = 0.010) odds of MV96 (Table 6).

**Table 6:** Multivariable logistic regression for mechanical ventilation >96 h

Factor	OR MV96 (95% CI)	*P*-value
Age		
0–9	6.45 (0.64–65.61)	0.115
10–19	0 (0–inf)	0.987
20–34	Ref.	–
35–49	1.92 (0.87–4.24)	0.108
50–64	2.63 (1.17–5.94)	**0.020**
65+	1.15 (0.24–5.53)	0.864
Procedures and diagnoses		
Mitral valve	1.54 (0.68–3.51)	0.300
Aortic valve	1.15 (0.52–2.55)	0.722
Coronary artery	0.93 (0.26–3.34)	0.911
TADR	2.19 (1.21–3.97)	**0.010**

CI: confidence interval; MV96: mechanical ventilation >96 h; OR: odds ratio; TADR: thoracic and thoraco-abdominal dissections and ruptures.

Bold values denote statistical significance.

### Length of stay

Median LOS was 10 (IQR 7–16) days (Table 7). Medicaid-insured discharges had the longest LOS [14 (IQR 9–21) days, *P* = 0.019]. Hospital volume was associated with LOS (*P* < 0.001), with the high-volume tertile [11 (IQR 7–16) days] having the longest LOS, followed by the mid-volume tertile [9 (IQR 5–13) days] then the low-volume tertile [8 (IQR 5–11 days)]. Discharges with TADR had longer LOS compared to those without [11 (IQR 9–19) vs 9 (IQR 6–15) days, *P* < 0.001]. These associations persisted after adjustment with 22.8% (95% CI 5–43.6%, *P* = 0.010) longer LOS among those 50–64 years and 181.3% (95% CI 49.2–430.6%, *P* = 0.001) among the 0–9 year age groups compared to the 20–34 year age group, Medicaid with 20.9% (95% CI 2.2–43.1%, *P* = 0.027) longer LOS relative to private insurance, the high-volume tertile having longer LOS [48% (95% CI 23.3–77.8%, *P* < 0.001) compared to the low-volume tertile, and TADR with 15.0% (95% CI 2.4–29.1%, *P* = 0.019)] longer LOS (Table 8).

**Table 7:** Univariate outcomes: length of stay^a^

Factor	Median LOS, days (IQR)	*P*-value
Total	10 (7–16)	–
Demographics		
Female	10.5 (7–16)	0.120
Male	10 (7–15)	
White	10 (7–16)	0.434
Non-White	10.5 (7–15)	
Hispanic	10 (7–15)	0.809
Non-Hispanic	10 (7–16)	
Age (years)		
0–9	13.5 (11.75–21.75)	**<0.001**
10–19	6 (5–7.5)	
20–34	9 (7–13)	
35–49	11 (7.5–16.5)	
50–64	12 (8.5–18)	
65+	11 (7.25–16.5)	
Type of insurance		
Private	10 (7–15)	**0.019**
Uninsured	10 (8–15)	
Medicare	10 (7–15.25)	
Medicaid	14 (9–21)	
Other	10 (8–13.75)	
Tertiles by hospital volume		
Low volume	8 (5–11)	**<0.001**
Mid volume	9 (5–13)	
High volume	11 (7–16)	
Risk factors		
Hypertension	11 (7–16)	0.130
No hypertension	9.5 (6.75–15)	
Atherosclerosis	12 (10.5–17)	0.080
No atherosclerosis	10 (7–16)	
Lipid disorders	10 (7–16)	0.707
No lipid disorders	10 (7–16)	
Diabetes mellitus	11 (8–14.75)	0.380
No diabetes mellitus	10 (7–16)	
Tobacco use	11 (7–17)	0.398
No tobacco use	10 (7–15)	
Bicuspid aortic valve	6.5 (4.75–8.25)	**<0.001**
Non-bicuspid aortic valve	10 (7–16)	
Procedure		
Mitral valve	11 (7–15)	0.988
No mitral valve involvement	10 (7–16)	
Aortic valve	10 (7–14.25)	0.290
No aortic valve involvement	10 (7–16)	
Coronary artery	10 (7–14)	0.752
No coronary involvement	10 (7–16)	
Endovascular	9 (6–13)	0.099
Open	10 (7–16)	
Principal diagnosis		
TADR	11 (9–19)	**<0.001**
No TADR	9 (6–15)	

a Excludes in-hospital mortalities.

IQR: interquartile range; LOS: length of stay; TADR: thoracic and thoraco-abdominal dissections and ruptures.

Bold values denote statistical significance.

**Table 8:** Multivariable linear regression of percent change in length of stay

Factor	% LOS difference (95% CI)^a^	*P*-value
Demographics		
Female	3.4 (–7.3 to 15.2)	0.551
White	–4 (–14.8 to 8.2)	0.505
Hispanic	1.6 (–11.6 to 16.8)	0.825
Age (years)		
0–9	181.3 (49.2 to 430.6)	**0.001**
10–19	–25.9 (–41.5 to –6.2)	**0.013**
20–34	Ref.	–
35–49	19.5 (4 to 37.3)	**0.012**
50–64	22.8 (5 to 43.6)	**0.010**
65+	24.3 (–3.7 to 60.4)	0.095
Insurance		
Private	Ref.	–
Uninsured	0.8 (–17.4 to 23)	0.938
Medicare	21.2 (–2.6 to 50.9)	0.086
Medicaid	20.9 (2.2 to 43.1)	**0.027**
Other	12.6 (–14.4 to 48.2)	0.396
Risk factors		
Hypertension	–6.8 (–17 to 4.7)	0.238
Lipid disorders	–9.5 (–23.8 to 7.5)	0.257
Diabetes mellitus	6.2 (–17.8 to 37.3)	0.646
Tobacco	1.5 (–12.8 to 18.2)	0.849
Atherosclerosis	26.2 (–16.1 to 89.7)	0.264
Bicuspid aortic valve	–22.9 (–45.5 to 9)	0.141
Hospital tertiles		
Low volume	Ref.	–
Mid volume	22.9 (–2.1 to 54.3)	0.076
High volume	48 (23.3 to 77.8)	**<0.001**
Procedure		
Mitral valve	14.4 (–2.4 to 34.1)	0.099
Aortic valve	7.0 (–7.4 to 23.6)	0.359
Coronary artery	–14.8 (–33.5 to 9.2)	0.207
Endovascular	–11.4 (–29.4 to 11.1)	0.293
Diagnosis		
TADR	15.0 (2.4 to 29.1)	**0.019**
In-hospital mortality	–44.1 (–56.6 to –27.9)	**<0.001**

a Additionally adjusted for year.

CI: confidence interval; LOS: length of stay; OR: odds ratio; TADR: thoracic and thoraco-abdominal dissections and rupture.

Bold values denote statistical significance.

## DISCUSSION

We present contemporary outcomes of TAI performed in MFS patient discharges over the course of 11 years in a large, statewide database. To the authors’ knowledge, this is the largest cohort of MFS hospitalizations reported, with 4641 MFS discharges identified undergoing 644 TAI [22]. Unlike other databases, such as National Registry of Genetically Triggered Thoracic Aortic Aneurysms or International Registry of Acute Aortic Dissection, this study addresses the TAI outcomes of all MFS hospitalizations, not restricted to those with TADR [16, 17].

TAIs are a frequent occurrence, documented in ∼14% of MFS discharges and confer significant morbidity and an in-hospital mortality rate of 4.7%. This mortality rate is similar to previous reports on 30-day mortality for patients undergoing TAI in the general population, suggesting MFS may confer little additional risk of in-hospital mortality [23, 24].

### Age

Life expectancy in MFS has improved significantly from 30 years to 60+ years at experienced centres [3, 25]. As this population ages, the potential need for TAI is substantial: 30% of all TAI occurred in discharges >50 years in this study. Age was associated with increased morbidity with increased ARF, MV96 and longer LOS among 35–49 and 50–64 year groups. Identifying those with increased risk of undergoing TAI and increased risk of morbidity and mortality post-TAI may help develop surveillance guidelines and strengthen focused care teams to improve their perioperative care.

### Thoracic or thoraco-abdominal aortic dissections and rupture

TADR is a well-known and devastating consequence of MFS, being a persistent source of mortality in MFS historically and contemporarily [3, 26]. This study agrees that TADR persists as a significant problem, with 4.8% of all MFS discharges having a principal diagnosis of TADR and 34.6% of TAI occurring in the setting of TADR.

As expected, TADR was associated with many adverse outcomes (ARF, MV96 and prolonged LOS). ARF is worrisome as a recent meta-analysis demonstrated acute kidney injury having increased risk of long-term mortality after cardiac surgery [27]. Preventing ARF among TADR should be a target for improving short-and-long-term morbidity. TADR had no associations with in-hospital mortality, but this is likely secondary to a mixture of acute, non-acute and historical TADR diagnoses, biasing these data towards the null. The high prevalence and considerable morbidity of TADR undergoing TAI indicate surveillance and primary prevention of TADR should continue to be a topic of study. Current guidelines recommend annual imaging of the aortic root in MFS, with increased frequency if aortic diameter >4.5 cm [28]. Given the high prevalence of TADR in this study cohort, and previous data suggesting aortic size >5.5 cm is not a good predictor of TADR, this suggests either greater adherence to guidelines is needed or revisiting surveillance measures is warranted [11, 12, 29].

### Procedural considerations

Despite the potential complexity of adding mitral or aortic valvular interventions during TAI, they were not associated with adverse outcomes. However, concomitant coronary artery interventions conferred 3.69-fold adjusted odds of in-hospital mortality—the only factor with this association—and 2.66-fold odds of ARF. The reason for increased risk with coronary interventions are unclear, especially without knowing the exact indications for coronary intervention. But given their substantial morbidity and mortality, TAI with coronary interventions should likely be performed in experienced centres.

Endovascular approach was not associated with adverse outcomes in this study, which is consistent with recent reports [14]. However, there were no LOS benefits or decreased morbidity as one might expect with a less invasive approach. Some guidelines recommend against endovascular approaches for those with genetic aortopathy except in bail-out situations, though they were released after this study period concluded [15]. The endovascular approach was potentially reserved for sicker patients not amenable to open repair, therefore not conferring improvement in LOS. With no specific associations with adverse outcomes found in this study, it may be a viable treatment option, at least in the short term, and should be a target for continued inquiry. These results must be interpreted with caution as this study cannot interpret the long-term consequences where continued aortic dilation inherent in MFS aortopathy may distort the repair.

Hospital volume revealed no difference in outcomes aside from a longer LOS in the high-volume tertile after adjustment. Despite the in-hospital mortality rate declining as centre volume increased, it did not amount to statistical significance. This likely reflects the increased complexity at these higher-volume specialty centres, though the small number of hospitals performing the majority of TAI in this study may introduce centre-specific biases.

### Limitations

This study has the limitations of using an administrative, discharge dataset. The sample is a hospitalized population, not the general MFS population. The unit of measure is discharges, not patients, so patients could be represented multiple times. Similarly, previous surgical and medical history are not able to be ascertained, including defined aspects of the Ghent criteria for MFS diagnosis [2]. Participating hospitals could misclassify or omit diagnoses or procedures, impacting analyses. ICD-9 codes lack granularity to distinguish thoracic and thoraco-abdominal location of aortic aneurysms or TADR; therefore, they were grouped together, which has significant implications on distinguishing between Stanford A vs B dissections. Similarly, it was impossible to discern between acute vs chronic vs historical TADR diagnoses. The absence of imaging to characterize aortic morphology precludes full understanding of aortopathy of those undergoing TAI. Finally, we do not know the temporal relationship between TAI and ICD-9/10 diagnosis codes; however, the median overall LOS was 10 (IQR 7–16) days and the median postoperative LOS was 9 (6–14) days, indicating that many diagnoses like MV96 likely occurred after the TAI.

## CONCLUSIONS

Over the past decade, TAIs remain frequent in the hospitalized MFS population. TAI are associated with significant morbidity, and conferred an in-hospital mortality rate of 4.7%. Concomitant coronary artery interventions are independently associated with increased risk of death and TADR is associated with higher rates of important morbidities. The high prevalence of TADR is a potential target for improvement in imaging surveillance, adherence to treatment guidelines, and preventative management of aortopathy in the MFS population.

## Supplementary Material

ivad128_Supplementary_Data

## Data Availability

The data in this article will be shared on reasonable request to the corresponding author. **Author contributions:** **Matthew F. Mikulski:** Conceptualization; Data curation; Formal analysis; Investigation; Methodology; Project administration; Validation; Visualization; Writing—original draft; Writing—review & editing. **Andrew Well:** Conceptualization; Data curation; Formal analysis; Investigation; Methodology; Supervision; Validation; Visualization; Writing—original draft; Writing—review & editing. **Carlos M. Mery:** Conceptualization; Validation; Writing—review & editing. **Gregory Johnson:** Conceptualization; Investigation; Writing—review & editing. **Erin A. Gottlieb:** Conceptualization; Supervision; Validation; Writing—review & editing. **Charles D. Fraser:** Conceptualization; Supervision; Validation; Writing—review & editing. **Ziv Beckerman:** Conceptualization; Formal analysis; Investigation; Methodology; Supervision; Validation; Visualization; Writing—original draft; Writing—review & editing. **Reviewer information:** Interdisciplinary CardioVascular and Thoracic Surgery thanks Florian Schoenhoff, Yutaka Okita, Gabriele Piffaretti and the other anonymous reviewer(s) for their contribution to the peer review process of this article.

## Notes

Presented at the AATS Aortic Symposium Workshop, Boston, MA, USA, 13–14 May 2022.

## ABBREVIATIONS

ARF: Acute renal failure
CI: Confidence interval
ICD: International Classification of Diseases
IQR: Interquartile range
LOS: Length of stay
MFS: Marfan syndrome
MV96: Mechanical ventilation >96 h
OR: Odds ratio
TADR: Thoracic or thoraco-abdominal aortic dissection or rupture
TAI: Thoracic aortic interventions
TIDD: Texas Inpatient Discharge Public Use File
TMCS: Temporary mechanical circulatory support
