# Supplementary material for: Outcomes of thoracic aortic interventions in Marfan syndrome in the state of Texas over 11 years
Source: Interdiscip Cardiovasc Thorac Surg. 2023 Aug 16;37(2):ivad128. doi: 10.1093/icvts/ivad128 (PMC10438956; doi:10.1093/icvts/ivad128)
Supplement: ivad128_Supplementary_Data [file ivad128_supplementary_data.docx]

**Supplementary material**

Supplemental Table 1: ICD Codes Utilized

| **Diagnoses and risk factors** | |
| --- | --- |
| Marfan Syndrome | 75982, Q8740, Q87410, Q87418, Q8742, Q8743 |
| Thoracic and Thoracoabdominal Aortic Dissections and Ruptures | 44101, 44103, I7101, I7103, 4411, 44110, 4416, 44160, I711, I715 |
| Hypertension | 4010, 4011, 4019, 40200, 40201, 40210, 40211, 40290, 40291, 40300, 40301, 40310, 40311, 40390, 40391, 40400, 40401, 40402, 40403, 40410, 40411, 40412, 40413, 40490, 40491, 40492, 40493, 40501, 40509, 40511, 40519, 40591, 40599, 642, I10, I110, I119, I120, I129, I130, I1310, I1311, I132, I150, I151, I152, I158, I159, I160, I161, I169, O10011, O10012, O10013, O10019, O1002, O1003, O10111, O10112, O10113, O10119, O1012, O1013, O10211, O10212, O10213, O10219, O1022, O1023, O10311, O10312, O10313, O10319, O1032, O1033, O10411, O10412, O10413, O10419, O1042, O1043, O10911, O10912, O10913, O10919,  O1092, O1093, O111, O112, O113, O114, O115, O119, O131, O132, O133, O134, O135, O139, O161, O162, O163, O164, O165, O169 |
| Atherosclerosis (Non-Coronary) | 4400, 4401, 4404, 4408, 4409, 44020, 44021, 44022, 44023, 44024, 44029, 44030, 44031, 44032, I700, I701, I70201, I70202, I70203, I70208, I70209, I70211, I70212, I70213, I70218, I70219, I70221, I70222, I70223, I70228, I70229, I70231, I70232, I70233, I70234, I70235, I70238, I70239, I70241, I70242, I70243, I70244, I70245, I70248, I70249, I7025, I70261, I70262, I70263, I70268, I70269, I70291, I70292, I70293, I70298, I70299, I70301, I70302, I70303, I70308, I70309, I70311, I70312, I70313, I70318, I70319, I70321, I70322, I70323, I70328, I70329, I70331, I70332, I70333, I70334, I70335, I70338, I70339, I70341, I70342, I70343, I70344, I70345, I70348, I70349, I7035, I70361, I70362, I70363, I70368, I70369, I70391, I70392, I70393, I70398, I70399, I70401, I70402, I70403, I70408, I70409, I70411, I70412, I70413, I70418, I70419, I70421, I70422, I70423, I70428, I70429, I70431, I70432, I70433, I70434, I70435, I70438, I70439, I70441, I70442, I70443, I70444, I70445, I70448, I70449, I7045, I70461, I70462, I70463, I70468, I70469, I70491, I70492, I70493, I70498, I70499, I70501, I70502, I70503, I70508, I70509, I70511, I70512, I70513, I70518, I70519, I70521, I70522, I70523, I70528, I70529, I70531, I70532, I70533, I70534, I70535, I70538, I70539, I70541, I70542, I70543, I70544, I70545, I70548, I70549, I7055, I70561, I70562, I70563, I70568, I70569, I70591, I70592, I70593, I70598, I70599, I70601, I70602, I70603, I70608, I70609, I70611, I70612, I70613, I70618, I70619, I70621, I70622, I70623, I70628, I70629, I70631, I70632, I70633, I70634, I70635, I70638, I70639, I70641, I70642, I70643, I70644, I70645, I70648, I70649, I7065, I70661, I70662, I70663, I70668, I70669, I70691, I70692, I70693, I70698, I70699, I70701, I70702, I70703, I70708, I70709, I70711, I70712, I70713, I70718, I70719, I70721, I70722, I70723, I70728, I70729, I70731, I70732, I70733, I70734, I70735, I70738, I70739, I70741, I70742, I70743, I70744, I70745, I70748, I70749, I7075, I70761, I70762, I70763, I70768, I70769, I70791, I70792, I70793, I70798, I70799, I708, I7090,  I7091 |
| Lipid Disorder | 2720, 2721, 2722, 2723, 2724, 2725, 2726, 2727, 2728, 2729, E784, E7800, E7801, E782, E7849, E785 |
| Diabetes mellitus | 25000, 25001, 25002, 25003, 25010, 25011, 25012, 25013, 25020, 25021, 25022, 25023, 25030, 25031, 25032, 25033, 25040, 25041, 25042, 25043, 25050, 25051, 25052, 25053, 25060, 25061, 25062, 25063, 25070, 25071, 25072, 25073, 25080, 25081, 25082, 25083, 25090, 25091, 25092, 25093, O24011, O24012, O24013, O24019, O2402, O2403, O24111, O24112, O24113, O24119, O2412, O2413, O24311, O24312, O24313, O24319, O2432, O2433, O24811, O24812, O24813, O24819, O2482, O2483, O24911, O24912, O24913, O24919, O2492, O2493, E0800, E0801, E0810, E0811, E0821, E0822, E0829, E08311, E08319, E083211, E083212, E083213, E083219, E083291, E083292, E083293, E083299, E083311, E083312, E083313, E083319, E083391, E083392, E083393, E083399, E083411, E083412, E083413, E083419, E083491, E083492, E083493, E083499, E083511, E083512, E083513, E083519, E083521, E083522, E083523, E083529, E083531, E083532, E083533, E083539, E083541, E083542, E083543, E083549, E083551, E083552, E083553, E083559, E083591, E083592, E083593, E083599, E0836, E0837X1, E0837X2, E0837X3, E0837X9, E0839, E0840, E0841, E0842, E0843, E0844, E0849, E0851, E0852, E0859, E08610, E08618, E08620, E08621, E08622, E08628, E08630, E08638, E08641, E08649, E0865, E0869, E088, E089, E1010, E1011, E1021, E1022, E1029, E10311, E10319, E103211, E103212, E103213, E103219, E103291, E103292, E103293, E103299, E103311, E103312, E103313, E103319, E103391, E103392, E103393, E103399, E103411, E103412, E103413, E103419, E103491, E103492, E103493, E103499, E103511, E103512, E103513, E103519, E103521, E103522, E103523, E103529, E103531, E103532, E103533, E103539, E103541, E103542, E103543, E103549, E103551, E103552, E103553, E103559, E103591, E103592, E103593, E103599, E1036, E1037X1, E1037X2, E1037X3, E1037X9, E1039, E1040, E1041, E1042, E1043, E1044, E1049, E1051, E1052, E1059, E10610, E10618, E10620, E10621, E10622, E10628, E10630, E10638, E10641, E10649, E1065, E1069, E108, E109, E1100, E1101, E1110, E1111, E1121, E1122, E1129, E11311, E11319, E113211, E113212, E113213, E113219, E113291, E113292, E113293, E113299, E113311, E113312, E113313, E113319, E113391, E113392, E113393, E113399, E113411, E113412, E113413, E113419, E113491, E113492, E113493, E113499, E113511, E113512, E113513, E113519, E113521, E113522, E113523, E113529, E113531, E113532, E113533, E113539, E113541, E113542, E113543, E113549, E113551, E113552, E113553, E113559, E113591, E113592, E113593, E113599, E1136, E1137X1, E1137X2, E1137X3, E1137X9, E1139, E1140, E1141, E1142, E1143, E1144, E1149, E1151, E1152, E1159, E11610, E11618, E11620, E11621, E11622, E11628, E11630, E11638, E11641, E11649, E1165, E1169, E118, E119, E1300, E1301, E1310, E1311, E1321, E1322, E1329, E13311, E13319, E133211, E133212, E133213, E133219, E133291, E133292, E133293, E133299, E133311, E133312, E133313, E133319, E133391, E133392, E133393, E133399, E133411, E133412, E133413, E133419, E133491, E133492, E133493, E133499, E133511, E133512, E133513, E133519, E133521, E133522, E133523, E133529, E133531, E133532, E133533, E133539, E133541, E133542, E133543, E133549, E133551, E133552, E133553, E133559, E133591, E133592, E133593, E133599, E1336, E1337X1, E1337X2, E1337X3, E1337X9, E1339, E1340, E1341, E1342, E1343, E1344, E1349, E1351, E1352, E1359, E13610, E13618, E13620, E13621, E13622, E13628, E13630, E13638, E13641, E13649, E1365, E1369, E138, E139 |
| Tobacco use | V1582, 3051, 64900, 64901, 64902, 64903, 64904, 98984, O99330, O99331, O99332, O99333, O99334, O99335, T65221A, T65221D, T65221S, T65222A, T65222D, T65222S, T65223A, T65223D, T65223S, T65224A, T65224D, T65224S, F17210, F17211, F17213, F17218, F17219, Z716, Z720 |
| Congenital Aortic Insufficiency  (Bicuspid Aortic Valve) | 7464, Q231 |
| **Procedures** | |
| Thoracic aortic interventions | 3804, 3814, 3834, 3922, 3973, 3923, 3805, 3815, 3835, 3845, 3865, 3885, 021W08A, 021W08B, 021W08D, 021W08F, 021W08G, 021W08H, 021W08P, 021W08Q, 021W08R, 021W08V, 021W09A, 021W09B, 021W09D, 021W09F, 021W09G, 021W09H, 021W09P, 021W09Q, 021W09R, 021W09V, 021W0AA, 021W0AB, 021W0AD, 021W0AF, 021W0AG, 021W0AH, 021W0AP, 021W0AQ, 021W0AR, 021W0AV, 021W0JA, 021W0JB, 021W0JD, 021W0JF, 021W0JG, 021W0JH, 021W0JP, 021W0JQ, 021W0JR, 021W0JV, 021W0KA, 021W0KB, 021W0KD, 021W0KF, 021W0KG, 021W0KH, 021W0KP, 021W0KQ, 021W0KR, 021W0KV, 021W0ZA, 021W0ZB, 021W0ZD, 021W0ZP, 021W0ZQ, 021W0ZR, 021W48A, 021W48B, 021W48D, 021W48P, 021W48Q, 021W48R, 021W49A, 021W49B, 021W49D, 021W49P, 021W49Q, 021W49R, 021W4AA, 021W4AB, 021W4AD, 021W4AP, 021W4AQ, 021W4AR, 021W4JA, 021W4JB, 021W4JD, 021W4JP, 021W4JQ, 021W4JR, 021W4KA, 021W4KB, 021W4KD, 021W4KP, 021W4KQ, 021W4KR, 021W4ZA, 021W4ZB, 021W4ZD, 021W4ZP, 021W4ZQ, 021W4ZR, 025W0ZZ, 025W3ZZ, 025W4ZZ, 027W04Z, 027W0DZ, 027W0ZZ, 027W34Z, 027W3DZ, 027W3ZZ, 027W44Z, 027W4DZ, 027W4ZZ, 02BW0ZX, 02BW0ZZ, 02BW3ZX, 02BW3ZZ, 02BW4ZX, 02BW4ZZ, 02CW0ZZ, 02CW3ZZ, 02CW4ZZ, 02HW00Z, 02HW02Z, 02HW03Z, 02HW0DZ, 02HW0YZ, 02HW30Z, 02HW32Z, 02HW33Z, 02HW3DZ, 02HW3YZ, 02HW40Z, 02HW42Z, 02HW43Z, 02HW4DZ, 02HW4YZ, 02LW3DJ, 02NW0ZZ, 02NW3ZZ, 02NW4ZZ, 02QW0ZZ, 02QW3ZZ, 02QW4ZZ, 02RW07Z, 02RW08Z, 02RW0JZ, 02RW0KZ, 02RW47Z, 02RW48Z, 02RW4JZ, 02RW4KZ, 02SW0ZZ, 02UW07Z, 02UW08Z, 02UW0JZ, 02UW0KZ, 02UW37Z, 02UW38Z, 02UW3JZ, 02UW3KZ, 02UW47Z, 02UW48Z, 02UW4JZ, 02UW4KZ, 02VW0CZ, 02VW0DZ, 02VW0EZ, 02VW0FZ, 02VW0ZZ, 02VW3CZ, 02VW3DZ, 02VW3EZ, 02VW3FZ, 02VW3ZZ, 02VW4CZ, 02VW4DZ, 02VW4EZ, 02VW4FZ, 02VW4ZZ |
| Endovascular thoracic aortic interventions | 3973, 021W48A, 021W48B, 021W48D, 021W48P, 021W48Q, 021W48R, 021W49A, 021W49B, 021W49D, 021W49P, 021W49Q, 021W49R, 021W4AA, 021W4AB, 021W4AD, 021W4AP, 021W4AQ, 021W4AR, 021W4JA, 021W4JB, 021W4JD, 021W4JP, 021W4JQ, 021W4JR, 021W4KA, 021W4KB, 021W4KD, 021W4KP, 021W4KQ, 021W4KR, 021W4ZA, 021W4ZB, 021W4ZD, 021W4ZP, 021W4ZQ, 021W4ZR, 025W3ZZ, 025W4ZZ, 027W34Z, 027W3DZ, 027W3ZZ, 027W44Z, 027W4DZ, 027W4ZZ, 02BW3ZX, 02BW3ZZ, 02BW4ZX, 02BW4ZZ, 02CW3ZZ, 02CW4ZZ, 02HW30Z, 02HW32Z, 02HW33Z, 02HW3DZ, 02HW3YZ, 02HW40Z, 02HW42Z, 02HW43Z, 02HW4DZ, 02HW4YZ, 02LW3DJ, 02NW3ZZ, 02NW4ZZ, 02QW3ZZ, 02QW4ZZ, 02RW47Z, 02RW48Z, 02RW4JZ, 02RW4KZ, 02UW37Z, 02UW38Z, 02UW3JZ, 02UW3KZ, 02UW47Z, 02UW48Z, 02UW4JZ, 02UW4KZ, 02VW3CZ, 02VW3DZ, 02VW3EZ, 02VW3FZ, 02VW3ZZ, 02VW4CZ, 02VW4DZ, 02VW4EZ, 02VW4FZ, 02VW4ZZ, 021X48A, 021X48B, 021X48D, 021X48P, 021X48Q, 021X48R, 021X49A, 021X49B, 021X49D, 021X49P, 021X49Q, 021X49R, 021X4AA, 021X4AB, 021X4AD, 021X4AP, 021X4AQ, 021X4AR, 021X4JA, 021X4JB, 021X4JD, 021X4JP, 021X4JQ, 021X4JR, 021X4KA, 021X4KB, 021X4KD, 021X4KP, 021X4KQ, 021X4KR, 021X4ZA, 021X4ZB, 021X4ZD, 021X4ZP, 021X4ZQ, 021X4ZR, 025X3ZZ, 025X4ZZ, 027X34Z, 027X3DZ, 027X3ZZ, 027X44Z, 027X4DZ, 027X4ZZ, 02BX3ZX, 02BX3ZZ, 02BX4ZX, 02BX4ZZ, 02CX3ZZ, 02CX4ZZ, 02HX30Z, 02HX32Z, 02HX33Z, 02HX3DZ, 02HX40Z, 02HX42Z, 02HX43Z, 02HX4DZ, 02NX3ZZ, 02NX4ZZ, 02QX3ZZ, 02QX4ZZ, 02RX47Z, 02RX48Z, 02RX4JZ, 02RX4KZ, 02UX37Z, 02UX38Z, 02UX3JZ, 02UX3KZ, 02UX47Z, 02UX48Z, 02UX4JZ, 02UX4KZ, 02VX3CZ, 02VX3DZ, 02VX3EZ, 02VX3FZ, 02VX3ZZ, 02VX4CZ, 02VX4DZ, 02VX4EZ, 02VX4FZ, 02VX4ZZ |
| Coronary artery interventions | 3603, 3604, 3606, 3607, 3609, 3610, 3611, 3612, 3613, 3614, 3615, 3616, 3617, 3619, 362, 3631, 3632, 3633, 3634, 3639, 3691, 3699, 0210083, 0210088, 0210089, 021008C, 021008F, 021008W, 0210093, 0210098, 0210099, 021009C, 021009F, 021009W, 02100A3, 02100A8, 02100A9, 02100AC, 02100AF, 02100AW, 02100J3, 02100J8, 02100J9, 02100JC, 02100JF, 02100JW, 02100K3, 02100K8, 02100K9, 02100KC, 02100KF, 02100KW, 02100Z3, 02100Z8, 02100Z9, 02100ZC, 02100ZF, 0210344, 02103D4, 0210444, 0210483, 0210488, 0210489, 021048C, 021048F, 021048W, 0210493, 0210498, 0210499, 021049C, 021049F, 021049W, 02104A3, 02104A8, 02104A9, 02104AC, 02104AF, 02104AW, 02104D4, 02104J3, 02104J8, 02104J9, 02104JC, 02104JF, 02104JW, 02104K3, 02104K8, 02104K9, 02104KC, 02104KF, 02104KW, 02104Z3, 02104Z8, 02104Z9, 02104ZC, 02104ZF, 0211083, 0211088, 0211089, 021108C, 021108F, 021108W, 0211093, 0211098, 0211099, 021109C, 021109F, 021109W, 02110A3, 02110A8, 02110A9, 02110AC, 02110AF, 02110AW, 02110J3, 02110J8, 02110J9, 02110JC, 02110JF, 02110JW, 02110K3, 02110K8, 02110K9, 02110KC, 02110KF, 02110KW, 02110Z3, 02110Z8, 02110Z9, 02110ZC, 02110ZF, 0211344, 02113D4, 0211444, 0211483, 0211488, 0211489, 021148C, 021148F, 021148W, 0211493, 0211498, 0211499, 021149C, 021149F, 021149W, 02114A3, 02114A8, 02114A9, 02114AC, 02114AF, 02114AW, 02114D4, 02114J3, 02114J8, 02114J9, 02114JC, 02114JF, 02114JW, 02114K3, 02114K8, 02114K9, 02114KC, 02114KF, 02114KW, 02114Z3, 02114Z8, 02114Z9, 02114ZC, 02114ZF, 0212083, 0212088, 0212089, 021208C, 021208F, 021208W, 0212093, 0212098, 0212099, 021209C, 021209F, 021209W, 02120A3, 02120A8, 02120A9, 02120AC, 02120AF, 02120AW, 02120J3, 02120J8, 02120J9, 02120JC, 02120JF, 02120JW, 02120K3, 02120K8, 02120K9, 02120KC, 02120KF, 02120KW, 02120Z3, 02120Z8, 02120Z9, 02120ZC, 02120ZF, 0212344, 02123D4, 0212444, 0212483, 0212488, 0212489, 021248C, 021248F, 021248W, 0212493, 0212498, 0212499, 021249C, 021249F, 021249W, 02124A3, 02124A8, 02124A9, 02124AC, 02124AF, 02124AW, 02124D4, 02124J3, 02124J8, 02124J9, 02124JC, 02124JF, 02124JW, 02124K3, 02124K8, 02124K9, 02124KC, 02124KF, 02124KW, 02124Z3, 02124Z8, 02124Z9, 02124ZC, 02124ZF, 0213083, 0213088, 0213089, 021308C, 021308F, 021308W, 0213093, 0213098, 0213099, 021309C, 021309F, 021309W, 02130A3, 02130A8, 02130A9, 02130AC, 02130AF, 02130AW, 02130J3, 02130J8, 02130J9, 02130JC, 02130JF, 02130JW, 02130K3, 02130K8, 02130K9, 02130KC, 02130KF, 02130KW, 02130Z3, 02130Z8, 02130Z9, 02130ZC, 02130ZF, 0213344, 02133D4, 0213444, 0213483, 0213488, 0213489, 021348C, 021348F, 021348W, 0213493, 0213498, 0213499, 021349C, 021349F, 021349W, 02134A3, 02134A8, 02134A9, 02134AC, 02134AF, 02134AW, 02134D4, 02134J3, 02134J8, 02134J9, 02134JC, 02134JF, 02134JW, 02134K3, 02134K8, 02134K9, 02134KC, 02134KF, 02134KW, 02134Z3, 02134Z8, 02134Z9, 02134ZC, 02134ZF, 02540ZZ, 02543ZZ, 02544ZZ, 0270046, 027004Z, 0270056, 027005Z, 0270066, 027006Z, 0270076, 027007Z, 02700D6, 02700DZ, 02700E6, 02700EZ, 02700F6, 02700FZ, 02700G6, 02700GZ, 02700T6, 02700TZ, 02700Z6, 02700ZZ, 0270346, 027034Z, 0270356, 027035Z, 0270366, 027036Z, 0270376, 027037Z, 02703D6, 02703DZ, 02703E6, 02703EZ, 02703F6, 02703FZ, 02703G6, 02703GZ, 02703T6, 02703TZ, 02703Z6, 02703ZZ, 0270446, 027044Z, 0270456, 027045Z, 0270466, 027046Z, 0270476, 027047Z, 02704D6, 02704DZ, 02704E6, 02704EZ, 02704F6, 02704FZ, 02704G6, 02704GZ, 02704T6, 02704TZ, 02704Z6, 02704ZZ, 0271046, 027104Z, 0271056, 027105Z, 0271066, 027106Z, 0271076, 027107Z, 02710D6, 02710DZ, 02710E6, 02710EZ, 02710F6, 02710FZ, 02710G6, 02710GZ, 02710T6, 02710TZ, 02710Z6, 02710ZZ, 0271346, 027134Z, 0271356, 027135Z, 0271366, 027136Z, 0271376, 027137Z, 02713D6, 02713DZ, 02713E6, 02713EZ, 02713F6, 02713FZ, 02713G6, 02713GZ, 02713T6, 02713TZ, 02713Z6, 02713ZZ, 0271446, 027144Z, 0271456, 027145Z, 0271466, 027146Z, 0271476, 027147Z, 02714D6, 02714DZ, 02714E6, 02714EZ, 02714F6, 02714FZ, 02714G6, 02714GZ, 02714T6, 02714TZ, 02714Z6, 02714ZZ, 0272046, 027204Z, 0272056, 027205Z, 0272066, 027206Z, 0272076, 027207Z, 02720D6, 02720DZ, 02720E6, 02720EZ, 02720F6, 02720FZ, 02720G6, 02720GZ, 02720T6, 02720TZ, 02720Z6, 02720ZZ, 0272346, 027234Z, 0272356, 027235Z, 0272366, 027236Z, 0272376, 027237Z, 02723D6, 02723DZ, 02723E6, 02723EZ, 02723F6, 02723FZ, 02723G6, 02723GZ, 02723T6, 02723TZ, 02723Z6, 02723ZZ, 0272446, 027244Z, 0272456, 027245Z, 0272466, 027246Z, 0272476, 027247Z, 02724D6, 02724DZ, 02724E6, 02724EZ, 02724F6, 02724FZ, 02724G6, 02724GZ, 02724T6, 02724TZ, 02724Z6, 02724ZZ, 0273046, 027304Z, 0273056, 027305Z, 0273066, 027306Z, 0273076, 027307Z, 02730D6, 02730DZ, 02730E6, 02730EZ, 02730F6, 02730FZ, 02730G6, 02730GZ, 02730T6, 02730TZ, 02730Z6, 02730ZZ, 0273346, 027334Z, 0273356, 027335Z, 0273366, 027336Z, 0273376, 027337Z, 02733D6, 02733DZ, 02733E6, 02733EZ, 02733F6, 02733FZ, 02733G6, 02733GZ, 02733T6, 02733TZ, 02733Z6, 02733ZZ, 0273446, 027344Z, 0273456, 027345Z, 0273466, 027346Z, 0273476, 027347Z, 02734D6, 02734DZ, 02734E6, 02734EZ, 02734F6, 02734FZ, 02734G6, 02734GZ, 02734T6, 02734TZ, 02734Z6, 02734ZZ, 02B40ZX, 02B40ZZ, 02B43ZX, 02B43ZZ, 02B44ZX, 02B44ZZ, 02C00Z6, 02C00ZZ, 02C03Z6, 02C03ZZ, 02C04Z6, 02C04ZZ, 02C10Z6, 02C10ZZ, 02C13Z6, 02C13ZZ, 02C14Z6, 02C14ZZ, 02C20Z6, 02C20ZZ, 02C23Z6, 02C23ZZ, 02C24Z6, 02C24ZZ, 02C30Z6, 02C30ZZ, 02C33Z6, 02C33ZZ, 02C34Z6, 02C34ZZ, 02C40ZZ, 02C43ZZ, 02C44ZZ, 02H00DZ, 02H00YZ, 02H03DZ, 02H03YZ, 02H04DZ, 02H04YZ, 02H10DZ, 02H10YZ, 02H13DZ, 02H13YZ, 02H14DZ, 02H14YZ, 02H20DZ, 02H20YZ, 02H23DZ, 02H23YZ, 02H24DZ, 02H24YZ, 02H30DZ, 02H30YZ, 02H33DZ, 02H33YZ, 02H34DZ, 02H34YZ, 02H400Z, 02H402Z, 02H403Z, 02H40DZ, 02H40JZ, 02H40KZ, 02H40MZ, 02H40NZ, 02H40YZ, 02H430Z, 02H432Z, 02H433Z, 02H43DZ, 02H43JZ, 02H43KZ, 02H43MZ, 02H43NZ, 02H43YZ, 02H440Z, 02H442Z, 02H443Z, 02H44DZ, 02H44JZ, 02H44KZ, 02H44MZ, 02H44NZ, 02H44YZ, 02N00ZZ, 02N03ZZ, 02N04ZZ, 02N10ZZ, 02N13ZZ, 02N14ZZ, 02N20ZZ, 02N23ZZ, 02N24ZZ, 02N30ZZ, 02N33ZZ, 02N34ZZ, 02N40ZZ, 02N43ZZ, 02N44ZZ, 02Q00ZZ, 02Q03ZZ, 02Q04ZZ, 02Q10ZZ, 02Q13ZZ, 02Q14ZZ, 02Q20ZZ, 02Q23ZZ, 02Q24ZZ, 02Q30ZZ, 02Q33ZZ, 02Q34ZZ, 02Q40ZZ, 02Q43ZZ, 02Q44ZZ, 0200ZZ, 0210ZZ, 02U007Z, 02U008Z, 02U00JZ, 02U00KZ, 02U037Z, 02U038Z, 02U03JZ, 02U03KZ, 02U047Z, 02U048Z, 02U04JZ, 02U04KZ, 02U107Z, 02U108Z, 02U10JZ, 02U10KZ, 02U137Z, 02U138Z, 02U13JZ, 02U13KZ, 02U147Z, 02U148Z, 02U14JZ, 02U14KZ, 02U207Z, 02U208Z, 02U20JZ, 02U20KZ, 02U237Z, 02U238Z, 02U23JZ, 02U23KZ, 02U247Z, 02U248Z, 02U24JZ, 02U24KZ, 02U307Z, 02U308Z, 02U30JZ, 02U30KZ, 02U337Z, 02U338Z, 02U33JZ, 02U33KZ, 02U347Z, 02U348Z, 02U34JZ, 02U34KZ |
| Mitral valve interventions | 3502, 3512, 3522, 3523, 3524, 3597, 024G072, 024G082, 024G0J2, 024G0K2, 025G0ZZ, 025G3ZZ, 025G4ZZ, 027G04Z, 027G0DZ, 027G0ZZ, 027G34Z, 027G3DZ, 027G3ZZ, 027G44Z, 027G4DZ, 027G4ZZ, 02BG0ZX, 02BG0ZZ, 02BG3ZX, 02BG3ZZ, 02BG4ZX, 02BG4ZZ, 02CG0ZZ, 02CG3ZZ, 02CG4ZZ, 02NG0ZZ, 02NG3ZZ, 02NG4ZZ, 02QG0ZE, 02QG0ZZ, 02QG3ZE, 02QG3ZZ, 02QG4ZE, 02QG4ZZ, 02RG07Z, 02RG08Z, 02RG0JZ, 02RG0KZ, 02RG37H, 02RG37Z, 02RG38H, 02RG38Z, 02RG3JH, 02RG3JZ, 02RG3KH, 02RG3KZ, 02RG47Z, 02RG48Z, 02RG4JZ, 02RG4KZ, 02UG07E, 02UG07Z, 02UG08E, 02UG08Z, 02UG0JE, 02UG0JZ, 02UG0KE, 02UG0KZ, 02UG37E, 02UG37Z, 02UG38E, 02UG38Z, 02UG3JE, 02UG3JH, 02UG3JZ, 02UG3KE, 02UG3KZ, 02UG47E, 02UG47Z, 02UG48E, 02UG48Z, 02UG4JE, 02UG4JZ, 02UG4KE, 02UG4KZ, 02VG0ZZ, 02VG3ZZ, 02VG4ZZ, 02WG07Z, 02WG08Z, 02WG0JZ, 02WG0KZ, 02WG37Z, 02WG38Z, 02WG3JZ, 02WG3KZ, 02WG47Z, 02WG48Z, 02WG4JZ, 02WG4KZ |
| Aortic valve interventions | 3501, 3505, 3506, 3511, 3521, 024F07J, 024F08J, 024F0JJ, 024F0KJ, 025F0ZZ, 025F3ZZ, 025F4ZZ, 027F04Z, 027F0DZ, 027F0ZZ, 027F34Z, 027F3DZ, 027F3ZZ, 027F44Z, 027F4DZ, 027F4ZZ, 02BF0ZX, 02BF0ZZ, 02BF3ZX, 02BF3ZZ, 02BF4ZX, 02BF4ZZ, 02CF0ZZ, 02CF3ZZ, 02CF4ZZ, 02NF0ZZ, 02NF3ZZ, 02NF4ZZ, 02QF0ZJ, 02QF0ZZ, 02QF3ZJ, 02QF3ZZ, 02QF4ZJ, 02QF4ZZ, 02RF07Z, 02RF08Z, 02RF0JZ, 02RF0KZ, 02RF37H, 02RF37Z, 02RF38H, 02RF38Z, 02RF3JH, 02RF3JZ, 02RF3KH, 02RF3KZ, 02RF47Z, 02RF48Z, 02RF4JZ, 02RF4KZ, 02UF07J, 02UF07Z, 02UF08J, 02UF08Z, 02UF0JJ, 02UF0JZ, 02UF0KJ, 02UF0KZ, 02UF37J, 02UF37Z, 02UF38J, 02UF38Z, 02UF3JJ, 02UF3JZ, 02UF3KJ, 02UF3KZ, 02UF47J, 02UF47Z, 02UF48J, 02UF48Z, 02UF4JJ, 02UF4JZ, 02UF4KJ, 02UF4KZ, 02WF07Z, 02WF08Z, 02WF0JZ, 02WF0KZ, 02WF37Z, 02WF38Z, 02WF3JZ, 02WF3KZ, 02WF47Z, 02WF48Z, 02WF4JZ, 02WF4KZ |
| **Outcomes** | |
| Mechanical ventilatory support >96 hours | 9672, 5A1955Z |
| Temporary Mechanical Circulatory Support or Extracorporeal Membrane Oxygenation | 3760, 3765, 3965, 5A02116, 5A0211D, 5A02216, 5A0221D, 02HA3RZ, 3768, 02HA0RS, 02HA3RS, 02HA4RS, 5A02116, 5A02216, 02HA0RZ, 02HA4RZ, 5A02216, 5A15223, 5A1522F, 5A1522G, 5A1522H |
| Acute Renal Failure | 5845, 5846, 5847, 5848, 5849, N170, N171, N172, N178, N179 |

Supplemental Table 2: Univariate analysis of demographics and baseline characteristics in relation to in-hospital mortality, acute renal failure, and mechanical ventilation >96 hours

|  | **Total** | **In-Hospital**  **Mortality**  ***n=30*** | **Alive**  ***n=614*** | ***p* value** |  | **ARF**  ***n=126*** | **No ARF**  ***n=518*** | ***p* value** |  | **MV96**  ***n=52*** | **No MV96**  ***n=592*** | ***p* value** |
| --- | --- | --- | --- | --- | --- | --- | --- | --- | --- | --- | --- | --- |
| **Demographics** |  |  |  |  |  |  |  |  |  |  |  |  |
| Female | 248 | 10(33.3%) | 238(38.8%) | 0.551 |  | 38(30.2%) | 210(40.5%) | **0.032** |  | 20(38.5%) | 228(38.5%) | 0.994 |
| White | 421 | 19(63.3%) | 402(65.5%) | 0.81 |  | 79(62.7%) | 342(66%) | 0.482 |  | 38(73.1%) | 383(64.7%) | 0.223 |
| Hispanic | 141 | 9(30.0%) | 132(21.5%) | 0.272 |  | 25(19.8%) | 116(22.4%) | 0.534 |  | 13(25%) | 128(21.6%) | 0.572 |
| **Age (years)** |  |  |  |  |  |  |  |  |  |  |  |  |
| 0-9 | 5 | 1(3.3%) | 4(0.7%) | 0.087 |  | 0(0%) | 5(1.0%) | **0.037** |  | 1(1.9%) | 4(0.7%) | 0.026 |
| 10-19 | 48 | 1(3.3%) | 47(7.7%) |  |  | 4(3.2%) | 44(8.5%) |  |  | 0(0%) | 48(8.1%) |  |
| 20-34 | 188 | 9(30.0%) | 179(29.2%) |  |  | 28(22.2%) | 160(30.9%) |  |  | 10(19.2%) | 178(30.1%) |  |
| 35-49 | 209 | 6(20.0%) | 203(33.1%) |  |  | 47(37.3%) | 162(31.3%) |  |  | 20(38.5%) | 189(31.9%) |  |
| 50-64 | 160 | 9(30.0%) | 151(24.6%) |  |  | 40(31.7%) | 120(23.2%) |  |  | 19(36.5%) | 141(23.8%) |  |
| 65+ | 34 | 4(13.3%) | 30(4.9%) |  |  | 7(5.6%) | 27(5.2%) |  |  | 2(3.8%) | 32(5.4%) |  |
| **Insurance** |  |  |  |  |  |  |  |  |  |  |  |  |
| Private | 437 | 18(60.0%) | 419(68.2%) | 0.483 |  | 81(64.3%) | 356(68.7%) | 0.712 |  | 34(65.4%) | 403(68.1%) | 0.068 |
| Uninsured | 53 | 4(13.3%) | 49(8.0%) |  |  | 12(9.5%) | 41(7.9%) |  |  | 2(3.8%) | 51(8.6%) |  |
| Medicare | 51 | 3(10.0%) | 48(7.8%) |  |  | 9(7.1%) | 42(8.1%) |  |  | 4(7.7%) | 47(7.9%) |  |
| Medicaid | 77 | 5(16.7%) | 72(11.7%) |  |  | 19(15.1%) | 58(11.2%) |  |  | 12(23.1%) | 65(11.0%) |  |
| Other | 26 | 0(0%) | 26(4.2%) |  |  | 5(4.0%) | 21(4.1%) |  |  | 0(0%) | 26(4.4%) |  |

Supplemental Table 3: Univariate analysis of temporary mechanical circulatory support

|  | Total | TMCS  *n=17* | No TMCS  *n=627* | *p* value |
| --- | --- | --- | --- | --- |
| Demographics |  |  |  |  |
| Female | 248 | 6(35.3%) | 242(38.6%) | 0.782 |
| White | 421 | 9(52.9%) | 412(65.7%) | 0.275 |
| Hispanic | 141 | 2(11.8%) | 139(22.2%) | 0.306 |
| Age (years) |  |  |  |  |
| 0-9 | 5 | 0(0%) | 5(0.8%) | 0.748 |
| 10-19 | 48 | 0(0%) | 48(7.7%) |  |
| 20-34 | 188 | 5(29.4%) | 183(29.2%) |  |
| 35-49 | 209 | 8(47.1%) | 201(32.1%) |  |
| 50-64 | 160 | 4(23.5%) | 156(24.9%) |  |
| 65+ | 34 | 0(0%) | 34(5.4%) |  |
| Insurance |  |  |  |  |
| Private | 437 | 13(76.5%) | 424(67.6%) | 0.91 |
| Uninsured | 53 | 2(11.8%) | 51(8.1%) |  |
| Medicare | 51 | 1(5.9%) | 50(8.0%) |  |
| Medicaid | 77 | 1(5.9%) | 76(12.1%) |  |
| Other | 26 | 0(0%) | 26(4.1%) |  |
| Hospital Tertile |  |  |  |  |
| Low-volume | 68 | 0(0%) | 68(10.8) | 0.374 |
| Mid--volume | 78 | 1(5.9%) | 77(12.3%) |  |
| High-volume | 498 | 16(94.1%) | 482(76.9%) |  |
| Co-morbidities |  |  |  |  |
| Hypertension | 416 | 12(70.6%) | 404(64.4%) | 0.601 |
| Atherosclerosis | 11 | 0(0%) | 11(1.8%) | 0.582 |
| Lipid disorders | 78 | 1(5.9%) | 77(12.3%) | 0.425 |
| Diabetes mellitus | 30 | 1(5.9%) | 29(4.6%) | 0.808 |
| Tobacco use | 100 | 6(35.3%) | 94(15.0%) | **0.023** |
| Bicuspid aortic valve | 16 | 0(0%) | 16(2.6%) | 0.505 |
| Procedures |  |  |  |  |
| Mitral valve | 88 | 3(17.6%) | 85(13.6%) | 0.628 |
| Aortic valve | 119 | 4(23.5%) | 115(18.3%) | 0.587 |
| Coronary artery | 33 | 3(17.6%) | 30(4.8%) | **0.018** |
| Endovascular | 42 | 0(0%) | 42(6.7%) | 0.27 |
| Diagnosis |  |  |  |  |
| TADR | 223 | 10(58.8%) | 213(34.0%) | **0.034** |
